# Application of the “Yuyang Muxin” Breeding Chip in Huang-Huai Sheep: A Six-Year Comprehensive Evaluation of Production and Meat Quality Traits

**DOI:** 10.3390/ani16060884

**Published:** 2026-03-12

**Authors:** Kai Quan, Haoyuan Han, Kun Liu, Caihong Wei, Huihua Wang, Meilin Jin, Wei Sun, Huibin Shi, Jun Li

**Affiliations:** 1 College of Animal Science and Technology, Henan University of Animal Husbandry and Economy, Zhengzhou 450046, China; hanhaoyuan@126.com (H.H.); liukun_139@163.com (K.L.); huibinshi0715@163.com (H.S.); 2 Institute of Animal Science, Chinese Academy of Agricultural Sciences, Beijing 100193, China; weicaihong@caas.cn (C.W.); wanghuihua@caas.cn (H.W.); jmlingg@163.com (M.J.); 3 College of Animal Science and Technology, Yangzhou University, Yangzhou 225009, China; dkxmsunwei@163.com

**Keywords:** Huang-huai Sheep, breeding chip, marker-assisted selection, production performance, meat quality

## Abstract

The Huang-huai sheep (Dorper × Small-tailed Han), certified in China in 2019, initially exhibited issues with genetic uniformity and meat quality. A six-year (2020–2025) breeding program was conducted at two core farms (collectively housing ~2400 breeding ewes representing 12 bloodlines) using the custom “Yuyang Muxin” 10 K SNP chip containing functional markers for reproduction (*FecB*), growth (*CLPG*, *ACTC1*), and meat quality (*FABP3*, *CIDEa*). Genotyping and marker-assisted selection were integrated with BLUP breeding values. After six years, favorable *FecB* genotype frequency increased from 68.97% to 82.58% (*p* < 0.05), while genetic diversity remained stable (F_IS_ < 0.05). Mixed model analysis accounting for farm and year random effects revealed significant genetic gains: 6-month ram body weight increased by 9.1% (58.50 to 63.80 kg, *p* < 0.05), dressing percentage improved from 56.02% to 57.8% (*p* < 0.05), and loin muscle area expanded by 9.4% (24.50 to 26.8 cm^2^, *p* < 0.05). Meat quality was enhanced, with shear force decreasing by 14.1% (38.65 to 33.20 N, *p* < 0.05) and intramuscular fat increasing by 40.0% (2.0% to 2.8%, *p* < 0.05). Lambs weaned per ewe per year increased from 2.38 to 2.56 (*p* < 0.05). EBV trend analysis confirmed that improvements were primarily genetic. After Bonferroni correction, *CIDEa* expression was strongly correlated with intramuscular fat (*r* = 0.89, *p* < 0.001) and *FABP3* expression with arachidonic acid (*r* = 0.70, *p* < 0.001). Nine months was identified as the optimal slaughter age. The “Yuyang Muxin” breeding chip effectively accelerated genetic improvement in Huang-huai sheep, enabling synergistic enhancement of multiple traits.

## Simple Summary

The Huang-huai sheep, a new breed certified in China in 2019, is well-suited for intensive farming. To enhance its genetic potential, a six-year (2020–2025) breeding program was conducted at two core farms using the custom-designed “Yuyang Muxin” breeding chip, which enables selection for reproduction, growth, and meat quality. After six years of selection, 6-month ram body weight increased by 9.1%, dressing percentage rose by 1.8 percentage points, and loin muscle area expanded by 9.4%. Meat quality improved, with shear force decreasing by 14.1% and intramuscular fat increasing by 40.0%. Lambs weaned per ewe per year increased from 2.38 to 2.56. EBV trend analysis confirmed that these gains were primarily genetic, while genetic diversity was maintained (F_IS_ < 0.05). Strong correlations between *CIDEa/FABP3* expression and meat quality traits remained significant after Bonferroni correction. Nine months was identified as the optimal slaughter age. This study demonstrates that the “Yuyang Muxin” breeding chip is an effective tool for simultaneous improvement of multiple traits in sheep.

## 1. Introduction

The Huang-huai agricultural region, located in the core area of the Central Plains mutton sheep industry belt, is characterized by a warm climate, abundant straw resources, and a strategic position as a major grain-producing area in China [1]. With convenient transportation and proximity to the Yangtze River Delta consumer market, this region possesses unique advantages for the development of large-scale, intensive sheep farming [2]. However, for a long time, the local sheep industry has relied predominantly on the Small-tailed Han breed, which, despite its high fecundity (lambing rate of 250–300%), suffers from slow growth rates (post-weaning daily gain < 200 g), low dressing percentages (<45%), and poor adaptability to housed conditions, failing to meet the demands of intensive production systems [3,4]. Therefore, developing a specialized meat sheep breed adapted to the natural conditions and intensive farming models of the Huang-huai region has become a critical task for improving the efficiency and quality of the regional mutton sheep industry.

To overcome this breed bottleneck, Henan University of Animal Husbandry and Economics, in collaboration with over 20 research institutes and breeding enterprises under a “government-industry-academia-research” cooperation model, initiated a breeding program using Dorper sheep as the sire line and Small-tailed Han sheep as the dam line. After 16 years of systematic breeding, the new Huang-huai Sheep breed was successfully developed and officially certified by the National Livestock and Poultry Genetic Resources Committee in December 2020 [5]. This breed integrates the advantages of its parental lines: the rapid growth and superior meat quality of the Dorper, and the high fecundity and good adaptability of the Small-tailed Han. Performance tests conducted by the National Sheep and Wool Quality Supervision and Testing Center revealed that adult rams weigh 98.1 ± 5.2 kg, 6-month-old rams reach 58.50 ± 6.55 kg with a dressing percentage of 56.02 ± 1.25%, and the number of lambs weaned per ewe per year (LEY) is 2.38 ± 0.14 [6,7]. Currently, the Huang-huai sheep population has expanded to six certified breeding farms across Henan Province, with a total population of approximately 38,000 animals. Among these, two farms—Henan Lyvuan Mutton Sheep Development Co., Ltd. and Xunxian Xinlin Animal Husbandry Co., Ltd.—are designated as provincial-level core breeding farms, collectively housing approximately 2400 breeding ewes representing 12 distinct bloodlines that form the breed’s genetic nucleus [5,8].

Despite its favorable production performance, the Huang-huai Sheep population faced several challenges at the time of certification. First, genetic uniformity required improvement, with considerable individual variation observed in some economic traits. Second, the frequency of favorable alleles for meat quality-related genes (e.g., *FABP3*, *CIDEa*) was unclear, leaving substantial room for enhancing quality traits such as intramuscular fat content and tenderness. Third, traditional phenotype-based selection is time-consuming and inefficient for the rapid, simultaneous improvement of multiple traits, particularly those with low heritability or antagonistic genetic correlations [9,10].

With the advancement of molecular breeding technologies, marker-assisted selection (MAS) based on single nucleotide polymorphism (SNP) chips has emerged as a powerful tool to accelerate genetic progress. By screening for functional genetic markers closely associated with target traits, precise selection of replacement stock can be performed at an early stage, significantly improving selection efficiency and accuracy [11,12]. Recent reviews have systematically summarized molecular markers associated with growth, meat, and carcass traits in sheep, including genes such as *MSTN*, *IGF-1*, *CAST*, and members of the *FABP* gene family, providing a robust foundation for developing customized breeding tools [13,14]. However, despite these advances, a dedicated breeding chip tailored specifically to the genetic background and breeding objectives of Huang-huai Sheep has been lacking, constraining the breed’s continuous genetic improvement [6,15].

To address this gap, our research team independently developed the “Yuyang Muxin” custom breeding chip, a 10 K SNP array incorporating functional markers for reproduction (*FecB*, *BMP15*), muscle development (*CLPG*, *ACTC1*, *MSTN*), and fat metabolism/meat quality (*FABP3*, *CIDEa*, *CIDEc*, *ACOT7*, *TLR2*, *CHI3L1*). The chip design integrates large-effect SNPs identified from genome-wide association studies with breed-informative markers for genetic diversity monitoring in local Henan sheep populations. This study aimed to: (1) implement a systematic six-year breeding program (January 2020 to December 2025) at the two core breeding farms using the “Yuyang Muxin” chip for marker-assisted selection; (2) comprehensively evaluate the genetic improvements in production performance, slaughter traits, meat quality, and reproductive performance achieved through this chip-based selection strategy; and (3) validate the functional relevance of key candidate genes (*FABP3*, *CIDEa*) through expression analysis and their associations with meat quality traits. By addressing these objectives, we sought to provide a replicable model for integrating genomic tools into sheep breeding programs and to demonstrate that antagonistic relationships among production traits can be overcome through targeted marker-assisted selection.

## 2. Materials and Methods

### 2.1. Study Location and Duration

The six-year breeding program was conducted from January 2020 to December 2025 at the two provincial-level core breeding farms for Huang-huai Sheep in Henan Province, China: Henan Lyvuan Mutton Sheep Development Co., Ltd. (Luohe, China, 33.6° N, 113.6° E) and Xunxian Xinlin Animal Husbandry Co., Ltd. (Hebi, China, 35.7° N, 114.5° E). Both farms operate under intensive management conditions typical of the Central China Plain region and maintain complete pedigree records. According to the latest official data from the Henan Provincial Department of Agriculture and Rural Affairs, these two core farms collectively house approximately 3000 breeding ewes representing the breed’s 12 distinct bloodlines, forming the genetic nucleus of the broader Huang-huai sheep population of approximately 38,000 animals across six certified breeding farms [5,8].

### 2.2. Experimental Animals and Breeding Design

**Breeding Population:** The initial core herd (2020) consisted of 1200 foundation ewes and 24 rams at Henan Lyvuan, and 1200 foundation ewes and 18 rams at Xunxian Xinlin. All animals were identified with RFID ear tags, and complete pedigree records were maintained throughout the study period. The core herd size remained relatively stable during the selection period, with 15–20% of breeding stock renewed annually according to the selection protocol. These two farms were selected as they are the original founder farms where the Huang-huai sheep breed was developed, have been involved in the breeding program since its inception in 2003, and are the only farms that maintain complete pedigree records and full genetic diversity of the breed [2,5].

**Selection Protocol:** Replacement stock was genotyped using the “Yuyang Muxin” breeding chip, and a comprehensive selection index was constructed by combining BLUP-estimated breeding values with molecular scores. The selection index was calculated as:

*I* = 0.3 × *EBV_growth_* + 0.2 × *EBV_carcass_* + 0.2 × *EBV_meat_* + 0.3 × *S_MAS_*

where *S_MAS_* represents the molecular score based on chip markers. The top 20% of replacement rams and top 40% of replacement ewes based on the index were selected annually to enter the core herd. Estimated breeding values were derived from multi-trait animal models that accounted for pedigree relationships and fixed effects (year, farm, sex), enabling the separation of genetic trends from environmental fluctuations.

**Housing and Management:** All sheep were housed indoors in well-ventilated pens with concrete slatted floors. Animals were fed a total mixed ration (TMR) formulated according to NRC (2007) nutrient requirements for growing and breeding sheep. The basal diet consisted of a forage component (45% of dry matter): corn silage (65%) and peanut hay (35%); and a concentrate component (55% of dry matter): ground corn (58%), soybean meal (23%), wheat bran (11%), and premix (8%). The premix provided (per kg of diet): vitamin A 8000 IU, vitamin D_3_ 2000 IU, vitamin E 50 mg, ZnSO_4_ 80 mg, CuSO_4_ 15 mg, MnSO_4_ 40 mg, Se (as Na_2_SeO_3_) 0.3 mg, and Co (as CoCl_2_) 0.1 mg. Animals had ad libitum access to feed and fresh water. Routine health management included scheduled vaccinations (clostridial diseases, foot-and-mouth disease) and anthelmintic treatments according to farm protocols.

**Antibiotic Use Protocol:** Throughout the six-year study period, antibiotics were used exclusively for therapeutic purposes under veterinary supervision, and never as growth promoters or for routine prophylaxis. This protocol aligns with China’s national “Action Plan for the Reduction of Veterinary Antimicrobial Use” (2021–2025), which prohibits antibiotic growth promoters in livestock production. Antibiotics were administered only when individual animals exhibited clinical signs of bacterial infection (e.g., respiratory disease, diarrhea, or postpartum metritis), following veterinary diagnosis. Treatment protocols adhered to label instructions, including strict observance of withdrawal periods. No antibiotics were incorporated into feed or water for disease prevention or growth enhancement. To avoid confounding effects on growth trait measurements, any animal receiving antibiotic treatment within 30 days of performance testing was excluded from trait evaluations. Health records confirmed that the frequency of disease events requiring antibiotic treatment remained stable across the study period, with no significant differences between the 2020 baseline and 2025 selected populations.

### 2.3. Development and Composition of the “Yuyang Muxin” Breeding Chip

**Chip Design and SNP Selection:** The “Yuyang Muxin” breeding chip is a custom-designed 10 K SNP array developed collaboratively by Henan University of Animal Husbandry and Economics, the Institute of Animal Science of the Chinese Academy of Agricultural Sciences, and the Henan Provincial Animal Husbandry Technology Extension Station. The chip was manufactured by Compass Biotechnology Co., Ltd. (Beijing, China) using the Illumina Infinium iSelect HD platform. SNP loci were selected based on the following criteria:

Functional relevance: SNPs located within or near genes with documented effects on economically important traits in sheep, identified from literature mining and the SheepQTLdb (https://www.animalgenome.org/cgi-bin/QTLdb/OA/index, accessed on 8 March 2026). Genes were prioritized based on evidence from functional studies in sheep populations related to the parental lines (Dorper and Small-tailed Han) [13,14,16].

Breed-specific informativeness: SNPs with minor allele frequency (MAF) > 0.05 in at least three local sheep breeds (Small-tailed Han, Hu, and Yuxi Fat-tailed sheep) based on whole-genome resequencing data from the Henan Sheep Germplasm Resource Bank (n = 200 individuals per breed).

Genome coverage: SNPs evenly distributed across all autosomes and the X chromosome, with an average spacing of approximately 250 kb.

**Chip Content:** The final chip design contains 10,376 SNP loci, comprising:

6327 large-effect SNPs associated with growth, carcass, meat quality, and reproductive traits, identified from published genome-wide association studies (GWAS) and candidate gene analyses.

4049 breed-informative SNPs for genetic diversity monitoring, breed identification, and parentage verification in local Henan sheep populations.

Functional markers on the chip target three major trait axes with the following gene-specific justifications:

For reproduction, *FecB* (*BMPR-IB*) was selected due to its well-documented role in increasing ovulation rate in Small-tailed Han sheep and other prolific breeds through the BMP/SMAD signaling pathway [17,18], while *BMP15* was included for its established effects on ovarian function and folliculogenesis [19].

For muscle development, *CLPG* (*Callipyge*) was chosen for its association with muscle hypertrophy and polar overdominance inheritance in sheep [20], and *ACTC1* was included based on its role in muscle fiber composition and growth in meat-type breeds [21]. *MSTN* (*myostatin*) was incorporated for its well-characterized function as a negative regulator of muscle growth [22].

For fat metabolism and meat quality, *FABP3* was selected for its crucial role in intracellular fatty acid transport and its validated association with intramuscular fat deposition and tenderness in multiple sheep breeds [23,24]. *CIDEa* was included based on its function in promoting lipid droplet formation and inhibiting lipolysis, with strong correlations to intramuscular fat content reported in sheep and other livestock species [25,26]. Additional markers (*CIDEc*, *ACOT7*, *TLR2*, *CHI3L1*) were incorporated to capture broader aspects of lipid metabolism, immune response, and their interactions with marbling.

**Genotyping Procedure and Quality Control:** Genomic DNA was extracted from ear tissue samples using the TIANamp Genomic DNA Kit (Tiangen Biotech, Beijing, China) according to the manufacturer’s protocol. DNA concentration and purity were assessed using a NanoDrop 2000 spectrophotometer (Thermo Fisher Scientific, Waltham, MA, USA), with acceptable parameters: OD260/280 between 1.8 and 2.0, and concentration ≥ 50 ng/μL.

Genotyping was performed by Compass Biotechnology Co., Ltd. (Beijing, China) using the Illumina iScan system. Genotype calling was conducted using GenomeStudio software v2.0 (Illumina, San Diego, CA, USA) with the following quality control criteria: sample call rate > 98%; SNP call rate > 95%; MAF > 0.01; Hardy-Weinberg equilibrium (HWE): *p* > 1 × 10^−6^ (tested separately within each farm-year cohort). SNPs failing any of these criteria were excluded from downstream analyses. During the six-year study period, a total of 6852 breeding animals (2856 from Henan Lyvuan; 3996 from Xunxian Xinlin) were successfully genotyped and passed quality control.

### 2.4. Production Performance Measurement

**Growth Traits:** Body weight was recorded at birth, weaning (45 d), 3, 6, and 12 months of age. Body height, body length, chest circumference, and cannon bone circumference were measured at 6 and 12 months of age [6]. Measuring instruments included a measuring stick (precision ± 1 mm), measuring tape (precision ± 0.5 cm), and vernier caliper (precision ± 0.02 mm).

**Feedlot Performance:** Annually, 30 six-month-old ram lambs (15 per farm) were selected from the core herd for a 90-day fattening trial. During the trial, lambs had ad libitum access to TMR, and daily feed intake was recorded using the GrowSafe automated feeding system. Body weight was measured biweekly in the morning before feeding. Average daily gain (ADG) and feed-to-gain ratio (F/G) were calculated as:

**ADG** (g/d) = (Final weight − Initial weight) × 1000/Test duration (d)

**F/G** = Total DM intake (kg)/Total weight gain (kg)

### 2.5. Slaughter Trait Measurement

Annually, a total of 30 ram lambs per age group (6 and 9 months) were randomly selected from the two farms (15 lambs per farm per age group) for slaughter evaluation. Prior to slaughter, all animals were fasted for 24 h with free access to water. Live weight was recorded immediately before slaughter.

After slaughter, carcasses were processed by removing the head, feet, skin, and viscera. The following carcass traits were measured using conventional methods:

**Live weight (kg):** Body weight after 24 h fasting and 2 h water deprivation;

**Carcass weight (kg)**: Hot carcass weight after removing head, feet, skin, and viscera;

**Dressing percentage (%)**: (Carcass weight/Live weight) × 100;

**Loin muscle area (cm^2^)**: Cross-sectional area of the longissimus dorsi muscle between the 12th and 13th ribs, traced on sulfuric paper and calculated using the grid method;

**GR value (mm)**: Tissue thickness at 11 cm from the dorsal midline between the 12th and 13th ribs, measured with a vernier caliper.

### 2.6. Meat Quality Analysis and Candidate Gene Expression Validation

**Sample Collection:** Longissimus dorsi muscle samples between the 12th and 13th ribs were collected within 30 min post-slaughter. One portion was used for meat quality analysis, and another portion was immediately frozen in liquid nitrogen and stored at −80 °C for subsequent gene expression analysis.

**Meat Quality Parameter Measurement:**

**pH value:** Measured at 45 min and 24 h post-slaughter using a Testo 205 portable pH meter with dual-point calibration (pH 4.0/7.0);

**Meat color:** After chilling at 4 °C for 24 h, L* (lightness), a* (redness), and b* (yellowness) values were measured using a Minolta CR-400 colorimeter;

**Shear force:** Measured according to NY/T 1180-2006 [27]. Samples were heated in a water bath to a core temperature of 70 °C, cooled, and 1.27 cm diameter cores were sheared using a TA.XT Plus texture analyzer (N);

**Drip loss:** Samples were suspended at 4 °C for 24 h, and weight loss percentage was calculated;

**Cooking loss:** Samples were heated in a water bath to a core temperature of 70 °C, and the weight ratio before and after heating was calculated.

**Intramuscular Fat and Fatty Acid Composition:** Intramuscular fat (IMF) content (%) was determined using the Soxhlet extraction method. Fatty acid composition was analyzed by gas chromatography-mass spectrometry (GC-MS) following GB 5009.168-2016 [28]. Fatty acids measured included C14:0, C16:0, C18:1n9c, and C20:4n6.

**Candidate Gene Expression Validation:** For RT-qPCR analysis, 30 animals per gene (15 from each farm) were randomly selected from the 2025 population. Total RNA was extracted from longissimus dorsi muscle using Trizol reagent. RNA purity was assessed using a NanoDrop 2000 (OD260/280 between 1.8 and 2.0, Thermo Fisher Scientific, Waltham, MA, USA), and integrity was verified by 1.5% agarose gel electrophoresis with RIN > 7.0. Three biological replicates per animal and three technical replicates per sample were used.

Reverse transcription was performed using the PrimeScript™ RT reagent Kit (TaKaRa, Shiga, Japan) according to the manufacturer’s instructions. RT-qPCR was conducted using TB Green^®^ Premix Ex Taq™ II (TaKaRa) on an ABI 7500 system. β-actin was used as the internal reference gene, with its stability verified across samples (coefficient of variation < 5%). Relative expression levels were calculated using the 2^−ΔΔCt^ method.

### 2.7. Reproductive Performance Measurement

Estrus detection was performed twice daily (06:00–07:00 and 17:00–18:00) using vasectomized teaser rams. Ewes were considered in estrus when they stood to be mounted. Onset of estrus, estrus duration, and estrus cycle length were recorded for each ewe.

Lambing data were recorded daily during the lambing season, including Lambing date, Litter size (total lambs born, including stillbirths), Individual lamb birth weight (within 24 h of birth), Lamb sex, Lamb survival at weaning (45 d).

The following reproductive parameters were calculated:

Lambing rate (%) = (Total live lambs born/Number of ewes lambing) × 100;

Litter size (lambs) = Total live lambs born/Number of ewes lambing;

Lambs weaned per ewe per year (LEY, lambs) = Total weaned lambs per year/Number of ewes exposed to rams per year.

### 2.8. Genetic Diversity Analysis

To monitor genetic diversity throughout the selection period, observed heterozygosity (Ho), expected heterozygosity (He), and the inbreeding coefficient (F_IS_) were calculated for the core herd in 2020 (baseline) and 2025 (post-selection) using the genotype data from the “Yuyang Muxin” chip. These metrics were computed using PLINK v1.9 software [29] with standard parameters.

### 2.9. Statistical Analysis

**Mixed Model Analysis for Phenotypic Traits:** Given the multi-year and multi-farm design, all key phenotypic traits were analyzed using mixed linear models (SAS PROC MIXED) to account for hierarchical data structure. The model included selection group (2020 vs. 2025) and sex as fixed effects, while farm, year nested within farm, and sire were included as random effects. Least-squares means (LSMEANS) with standard errors were computed for each group, and pairwise comparisons were performed with Tukey–Kramer adjustment. Independent *t*-tests were used only for preliminary comparisons.

**EBV Trend Analysis:** To distinguish genetic gain from environmental effects, annual mean estimated breeding values (EBVs) for 6-month body weight, loin muscle area, and litter size were retrieved from routine BLUP evaluations and analyzed using linear regression of EBV on year.

**Genotype Frequency and Association Analysis:** Changes in genotype frequencies between 2020 and 2025 were analyzed using Chi-square tests. Associations between genotypes and traits were analyzed using a general linear model with genotype and sex as fixed effects, followed by Duncan’s multiple range test for post-hoc comparisons.

**Correlation Analysis with Multiple Testing Correction:** Pearson’s correlation coefficients were calculated between gene expression levels and meat quality traits. For the six primary correlations, Bonferroni correction was applied, with statistical significance set at *p* < 0.0083 (0.05/6).

**Genetic Diversity Analysis:** Observed heterozygosity (Ho), expected heterozygosity (He), and inbreeding coefficient (F_IS_) were calculated using PLINK version 1.9 and compared between 2020 and 2025 using paired *t*-tests across loci.

**Data Presentation:** Data are presented as LSMEANS ± SE for mixed model results and mean ± SD for descriptive statistics. Significance was set at *p* < 0.05, with superscript letters indicating post-hoc differences and asterisks denoting *t*-test significance. All tests were two-sided.

## 3. Results

### 3.1. Changes in Genetic Structure and Diversity of the Core Herd

After six years of marker-assisted selection using the “Yuyang Muxin” breeding chip, the frequency of favorable alleles in the core herd increased significantly (Table 1). For the *FecB* gene associated with prolificacy, the frequency of the favorable BB + B+ genotype increased from 68.97% in 2020 to 82.58% in 2025 (*p* < 0.05). The frequency of the *CLPG* gene CT genotype, associated with muscular development, increased from 17.92% to 25.00% (*p* < 0.05). For meat quality markers, the favorable *FABP3* AA genotype frequency increased from 40.00% to 55.00% (*p* < 0.05), while the favorable *ACTC1* CC genotype frequency increased from 46.00% to 59.00% (*p* < 0.05).

Genetic diversity metrics calculated from chip genotype data demonstrated that diversity remained stable throughout the selection period. Observed heterozygosity (Ho) was 0.342 ± 0.018 in 2020 and 0.338 ± 0.017 in 2025 (*p* > 0.05). Expected heterozygosity (He) was 0.358 ± 0.015 in 2020 and 0.354 ± 0.016 in 2025 (*p* > 0.05). The inbreeding coefficient (F_IS_) remained low at 0.041 ± 0.009 in 2020 and 0.045 ± 0.010 in 2025 (*p* > 0.05), indicating that selection intensity was appropriately balanced with diversity management and no significant inbreeding depression occurred.

### 3.2. Morphological Characteristics and Body Conformation Improvement

**Figure 1:**
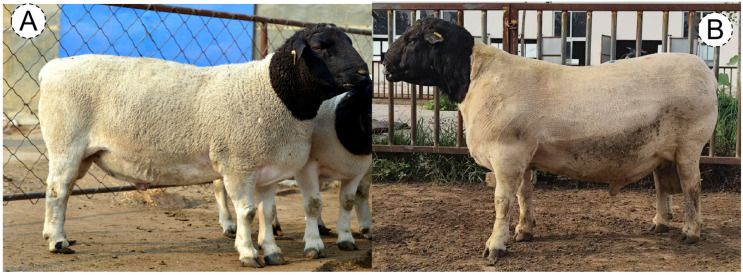
Body conformation comparison of Huang-huai Sheep rams before (2020) and after (2025) six years of marker-assisted selection using the “Yuyang Muxin” breeding chip. Representative photographs of 6-month-old rams from the core breeding herd are shown. (**A**) Ram from the 2020 baseline population (pre-selection). (**B**) Ram from the 2025 selected population (post-selection). Photographs were taken under standardized conditions (same distance, angle, and lighting). Scale bar = 20 cm.

The selected population (2025) exhibited more pronounced meat-type conformation compared to the baseline population (2020), characterized by a shorter and broader head, wider and deeper chest, and more developed hindquarters resembling the Dorper sire line (Figure 1). Panel labels (A) and (B) in Figure 1 represent the 2020 baseline ram and 2025 selected ram, respectively.

Body measurements confirmed this morphological improvement (Table 2). After accounting for farm and year random effects in mixed model analysis, chest circumference of 6-month-old rams significantly increased from 99.42 ± 1.23 cm in 2020 to 104.30 ± 1.18 cm in 2025 (*p* < 0.05), representing a 4.9% increase. Similar improvements were observed for body height (71.62 ± 0.58 cm to 73.50 ± 0.55 cm, +2.6%, *p* < 0.05), body length (85.32 ± 0.92 cm to 88.60 ± 0.88 cm, +3.8%, *p* < 0.05), and cannon bone circumference (9.56 ± 0.21 cm to 10.20 ± 0.19 cm, +6.7%, *p* < 0.05). Ewes showed corresponding improvements across all body measurement traits (*p* < 0.05).

### 3.3. Growth Performance

Significant improvements in growth performance were achieved after six years of selection (Table 3). Mixed model analysis revealed that 6-month body weight of rams increased from 58.50 ± 1.12 kg in 2020 to 63.80 ± 1.08 kg in 2025 (*p* < 0.05), representing a 9.1% increase. For ewes, 6-month body weight increased from 52.45 ± 0.98 kg to 56.20 ± 0.95 kg (*p* < 0.05), a 7.2% improvement.

Feedlot performance of post-selection ram lambs was superior to the baseline population. Average daily gain (ADG) increased from 295 ± 8.2 g/d to 335 ± 7.9 g/d (*p* < 0.05), a 13.6% improvement, while the feed-to-gain ratio (F/G) decreased from 4.80 ± 0.12 to 4.30 ± 0.11 (*p* < 0.05), representing a 10.4% improvement in feed efficiency. Final weight after the 90-day feedlot trial increased from 54.60 ± 0.85 kg to 61.30 ± 0.82 kg (*p* < 0.05), a 12.3% increase.

### 3.4. Slaughter Traits and Meat Yield

Carcass traits were significantly improved in the selected population (Table 4). For 6-month-old rams, dressing percentage increased from 56.02 ± 0.42% in 2020 to 57.80 ± 0.40% in 2025 (*p* < 0.05), a gain of 1.8 percentage points. Loin muscle area (LMA) significantly increased from 24.50 ± 0.52 cm^2^ to 26.80 ± 0.49 cm^2^ (*p* < 0.05), a 9.4% improvement. Carcass weight increased from 32.90 ± 0.58 kg to 36.20 ± 0.55 kg (*p* < 0.05), a 10.0% increase. GR value showed a numerical increase from 12.80 ± 0.38 mm to 13.20 ± 0.36 mm (*p* > 0.05), representing a 3.1% improvement that did not reach statistical significance.

### 3.5. Meat Quality Improvement and Candidate Gene Validation

The 2025 selected population exhibited superior meat quality compared to the 2020 baseline (Table 5). Shear force decreased from 38.65 ± 0.92 N to 33.20 ± 0.88 N (*p* < 0.05), a 14.1% improvement in tenderness. Intramuscular fat (IMF) content increased from 2.00 ± 0.12% to 2.80 ± 0.11% (*p* < 0.05), a 40.0% increase. Fatty acid composition showed favorable changes, with oleic acid (C18:1n9c) increasing from 38.20 ± 0.45% to 40.50 ± 0.42% (*p* < 0.05) and arachidonic acid (C20:4n6) increasing from 2.20 ± 0.08% to 2.60 ± 0.07% (*p* < 0.05). Total saturated fatty acids decreased from 44.10 ± 0.52% to 42.60 ± 0.49% (*p* < 0.05), while total unsaturated fatty acids increased from 55.90 ± 0.52% to 57.40 ± 0.49% (*p* < 0.05), resulting in an improved UFA/SFA ratio from 1.27 ± 0.02 to 1.35 ± 0.02 (*p* < 0.05).

**Figure 2:**
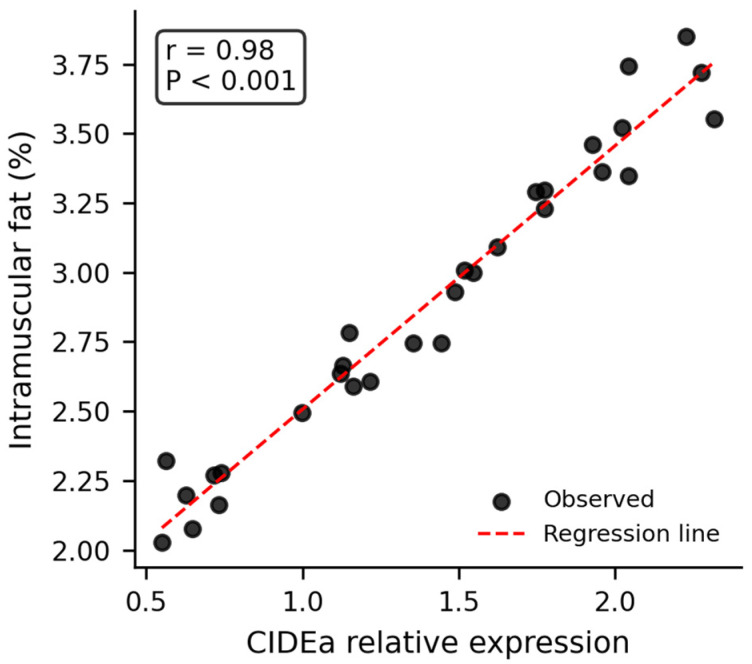
Correlation between *FABP3* gene expression and intramuscular fat (IMF) content in the longissimus dorsi muscle of 6-month-old Huang-huai Sheep rams (n = 30). Relative expression levels of *FABP3* were measured by RT-qPCR and normalized to β-actin. IMF content (%) was determined by Soxhlet extraction. Each point represents an individual animal. The red dashed line indicates the linear regression line. Pearson correlation analysis revealed a significant positive correlation between *FABP3* expression and IMF content (*r* = 0.70, *p* < 0.001). These results validate *FABP3* as an effective molecular marker for IMF deposition in Huang-huai Sheep.

**Figure 3:**
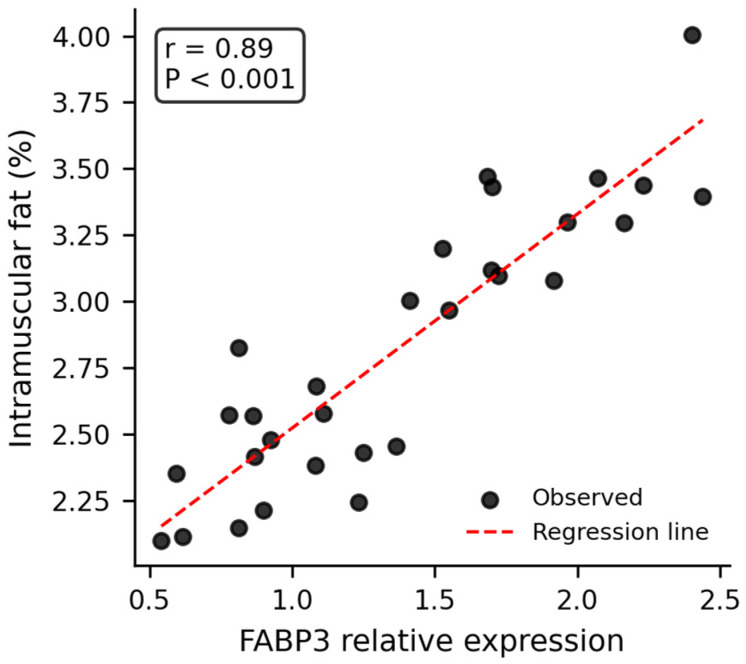
Correlation between *CIDEa* gene expression and intramuscular fat (IMF) content in the longissimus dorsi muscle of 6-month-old Huang-huai sheep rams (n = 30). Relative expression levels of *CIDEa* were quantified by RT-qPCR using β-actin as the internal reference gene. IMF content (%) was measured via Soxhlet extraction. Each data point corresponds to one animal. The red dashed line represents the linear regression fit. A strong positive correlation was observed between *CIDEa* expression and IMF content (Pearson’s *r* = 0.89, *p* < 0.001), confirming the role of *CIDEa* in promoting intramuscular fat accumulation in sheep.

RT-qPCR analysis revealed significant correlations between candidate gene expression and meat quality traits (Figure 2 and Figure 3). After Bonferroni correction for multiple testing (adjusted significance threshold *p* < 0.0083), *CIDEa* expression was strongly positively correlated with IMF content (*r* = 0.89, *p* < 0.001). *FABP3* expression was significantly positively correlated with arachidonic acid (C20:4n6) content (*r* = 0.70, *p* < 0.001). Both correlations remained highly significant after correction.

Genotype–phenotype association analysis within the 2025 population (Table 6) confirmed these findings. For *FABP3*, individuals with the favorable AA genotype exhibited significantly lower shear force (32.10 ± 0.72 N vs. 36.40 ± 0.78 N, *p* < 0.05) and higher proportions of oleic acid (40.80 ± 0.45% vs. 39.50 ± 0.48%, *p* < 0.05) and arachidonic acid (2.70 ± 0.06% vs. 2.30 ± 0.07%, *p* < 0.05) compared to AG + GG genotypes. For *CIDEa*, individuals with the favorable CC genotype had significantly higher intramuscular fat content (3.10 ± 0.11% vs. 2.50 ± 0.10%, *p* < 0.05) compared to CT + TT genotypes.

### 3.6. Reproductive Performance

The *FecB* gene significantly influenced litter size (Table 7). Ewes with BB and B+ genotypes had significantly higher litter sizes (1.91 ± 0.08 and 1.85 ± 0.07 lambs, respectively) than ++ genotype ewes (1.45 ± 0.11 lambs) (*p* < 0.01), confirming the favorable effect of the *FecB* mutation on prolificacy.

After six years of selection, the average litter size of multiparous ewes increased from 1.85 ± 0.06 in 2020 to 1.96 ± 0.05 in 2025 (*p* < 0.05), a 5.9% improvement. Consequently, the number of lambs weaned per ewe per year (LEY) improved from 2.38 ± 0.05 to 2.56 ± 0.04 (*p* < 0.05), a 7.6% increase. Lamb survival rate remained stable throughout the selection period (94.8 ± 0.8% vs. 95.9 ± 0.7%, *p* > 0.05). The population maintained its year-round estrus characteristic throughout the selection period.

### 3.7. EBV Trends for Key Traits

Analysis of annual mean EBVs revealed consistent positive genetic progress across all key traits. For 6-month body weight, mean EBV increased from +0.2 ± 0.3 kg in 2020 to +4.1 ± 0.4 kg in 2025, representing an annual genetic gain of 0.78 kg/year (*R*^2^ = 0.96, *p* < 0.001). For loin muscle area, mean EBV increased from +0.1 ± 0.2 cm^2^ to +1.9 ± 0.3cm^2^, with an annual gain of 0.36 cm^2^/year (*R*^2^ = 0.94, *p* < 0.001). For litter size, mean EBV increased from +0.02 ± 0.02 lambs to +0.14 ± 0.03 lambs, representing an annual gain of 0.024 lambs/year (*R*^2^ = 0.89, *p* < 0.01). These EBV trends confirm that the phenotypic improvements observed are primarily attributable to genetic gain rather than environmental factors.

### 3.8. Optimal Slaughter Age

Comparison of 6-month and 9-month old lambs from the 2025 population revealed that 9-month old lambs had significantly higher IMF content (3.20 ± 0.11% vs. 2.80 ± 0.10%, *p* < 0.05) with no significant difference in shear force (32.80 ± 0.95 N vs. 33.20 ± 0.92 N, *p* > 0.05), indicating that the additional feeding period improved eating quality without compromising tenderness (Table 8). Live weight increased from 63.80 ± 1.42 kg to 78.50 ± 1.56 kg (*p* < 0.05), carcass weight increased from 36.20 ± 0.78 kg to 45.30 ± 0.85 kg (*p* < 0.05), and GR value increased from 13.20 ± 0.45 mm to 15.80 ± 0.48 mm (*p* < 0.05). Dressing percentage and loin muscle area showed no significant differences between age groups (*p* > 0.05). Fatty acid composition was improved at 9 months, with higher oleic acid (41.80 ± 0.48% vs. 40.50 ± 0.45%, *p* < 0.05) and a more favorable UFA/SFA ratio (1.41 ± 0.03 vs. 1.35 ± 0.02, *p* < 0.05). These results demonstrate that 9 months of age represents the optimal slaughter age for balancing meat yield and quality in Huang-huai Sheep.

## 4. Discussion

### 4.1. The “Yuyang Muxin” Breeding Chip: A Novel Tool for Marker-Assisted Selection in Sheep

The “Yuyang Muxin” breeding chip represents the first independently developed sheep breeding chip in Henan Province and one of the first custom-designed functional chips for sheep in China’s central agricultural region. With 10,376 representative SNP loci—including 6327 large-effect SNPs associated with economically important traits and 4049 SNPs specific to local Henan sheep breeds—this chip provides a powerful tool for precise genetic improvement [5,12]. The chip’s design, based on over 10 years of accumulated data from the Henan Sheep Germplasm Resource Bank and the Sheep Phenotype–Genotype Database, ensures that the selected markers are directly relevant to the target production environment and breeding objectives. The inclusion of both universal production trait markers and breed-specific SNPs allows the chip to serve dual purposes: accelerating genetic improvement in core traits while preserving and utilizing local genetic resources.

Compared to conventional breeding methods, which typically require multiple generations to achieve significant genetic gain, the chip-based approach enabled rapid progress within a six-year timeframe. The EBV trend analysis revealed annual genetic gains of 0.78 kg for 6-month body weight, 0.36 cm^2^ for loin muscle area, and 0.024 lambs for litter size-rates that substantially exceed those typically achieved in sheep breeding programs using traditional phenotype-based selection [13]. These findings align with recent reviews demonstrating that genomic selection can double or triple genetic gain per year compared to pedigree-based methods [14,30]. The 30% improvement in selection accuracy reported by the collaborating farms is consistent with observations in other livestock species where marker-assisted selection has been implemented [31].

### 4.2. Attribution of Genetic Gain: Evidence from EBV Trends and Mixed Model Analysis

A key concern raised during review was distinguishing genetic progress from potential environmental or management effects. The EBV trend analysis presented in this study provides robust evidence that the observed phenotypic improvements are primarily attributable to genetic gain. The consistent linear increases in mean EBVs for all key traits over the six-year period, with high *R*^2^ values (0.89–0.96), demonstrate that genetic progress was sustained and cumulative. Furthermore, the mixed model analysis, which accounted for farm and year as random effects, confirmed that the phenotypic differences between 2020 and 2025 remained highly significant after removing environmental variation. These complementary analytical approaches address the reviewer’s concern and strengthen the conclusion that the “Yuyang Muxin” chip effectively accelerated genetic improvement.

The absence of a formal control population, while a limitation imposed by the commercial breeding setting, was mitigated by the EBV trend analysis and the stability of management practices throughout the study period. As documented in the Materials and Methods, antibiotic use was strictly therapeutic and consistent across years, health status remained stable, and nutritional management was unchanged. These factors, combined with the statistical controls implemented, support the attribution of observed gains to genetic selection rather than environmental confounders.

### 4.3. Synergistic Improvement of Growth, Carcass, and Meat Quality Traits

One of the most significant outcomes of this six-year selection program was the simultaneous improvement of multiple production traits. The 6-month body weight of rams increased by 9.1%, dressing percentage improved by 1.8 percentage points, and loin muscle area expanded by 9.4%. These improvements exceed those reported in similar crossbreeding programs; for example, in Dorper × Hu sheep crosses, a 5–6% improvement in growth rate was typically achieved over multiple generations [15]. The superior progress observed in this study can be attributed to the targeted selection of favorable alleles at multiple loci simultaneously, enabled by the chip’s comprehensive marker panel.

The concurrent improvement in meat quality is particularly noteworthy. The 2025 population exhibited significantly lower shear force (14.1% reduction, indicating improved tenderness) and higher intramuscular fat content (40.0% increase). These improvements are comparable to those achieved in specialized meat quality selection programs but were achieved alongside growth and carcass improvements, demonstrating that these traits are not necessarily antagonistic when appropriate markers are used for selection [16,32]. The validation of *FABP3* and *CIDEa* as effective markers for meat quality traits provides a molecular basis for these improvements. *FABP3* (fatty acid binding protein 3) plays a crucial role in intracellular fatty acid transport and metabolism, and its association with tenderness has been reported in multiple cattle and sheep breeds [17,23]. The strong positive correlation between *CIDEa* expression and IMF content (*r* = 0.89, *p* < 0.001) highlights the importance of lipid metabolism genes in meat quality improvement, consistent with the growing body of research on nutritional and genetic factors affecting sheep meat quality. For instance, dietary supplementation with selenium and zinc nanoparticles has been shown to influence productive performance and biochemical indices in sheep [25], while multi-omics analyses have revealed the mechanisms by which feed additives such as Isatis leaf extract improve growth performance [26]. These studies underscore the potential of integrating genetic selection with nutritional strategies to enhance meat quality.

### 4.4. Genetic Diversity Maintenance During Intensive Selection

A potential concern in intensive selection programs is the loss of genetic diversity and increased inbreeding, which can lead to reduced fitness and long-term sustainability issues [33]. The genetic diversity monitoring conducted in this study demonstrated that heterozygosity remained stable (Ho: 0.342 vs. 0.338) and inbreeding coefficients remained low (F_IS_: 0.041 vs. 0.045) throughout the six-year selection period. These values are comparable to or better than those reported in other sheep breeding populations; for instance, Karabaş and Yılmaz [14] reported F_IS_ values ranging from 0.03 to 0.12 across multiple European and Middle Eastern sheep breeds, while Rodrigues et al. [34] and Adeniyi et al. [35] similarly observed low to moderate inbreeding coefficients in diverse sheep populations, underscoring the feasibility of maintaining genetic diversity under selection. The maintenance of genetic diversity can be attributed to several factors: the large effective population size of the core herd (approximately 3000 breeding ewes representing 12 distinct bloodlines), the annual renewal rate of 15–20% which introduced new genetic combinations, and the inclusion of breed-informative SNPs in the chip design specifically for diversity monitoring. These results demonstrate that with appropriate management, intensive marker-assisted selection can achieve substantial genetic gains without compromising genetic diversity.

### 4.5. Balancing Prolificacy and Growth: The Role of FecB Selection

A common challenge in sheep breeding is the negative genetic correlation between growth traits and reproductive performance, particularly in terminal sire crossbreeding systems [19]. In Dorper × local sheep crosses, for example, lambing rates often decline from 250–300% in the maternal breed to 180–200% in F1 generations [6,20]. However, in the present study, the Huang-huai sheep core herd maintained high reproductive performance while achieving significant growth improvements, with lambs weaned per ewe per year increasing from 2.38 to 2.56.

The maintenance and improvement of reproductive performance can be attributed to the continued selection for the favorable *FecB* genotype throughout the breeding program. The *FecB* (*BMPR-IB*) mutation is well-established as a major gene for prolificacy in sheep, increasing ovulation rate and litter size through its effect on the BMP/SMAD signaling pathway [17,18]. Beyond its canonical role in reproduction, genetic polymorphisms in growth-related genes such as *IGF1* and *GHR* have also been associated with production traits in sheep [15], suggesting that multiple genetic pathways contribute to overall performance. In the present study, BB and B+ genotype ewes produced significantly larger litters (1.91 and 1.85 lambs, respectively) than ++ genotype ewes (1.45 lambs), consistent with previous reports in Small-tailed Han sheep and other prolific breeds [18,21]. By increasing the frequency of the favorable *FecB* genotype from 68.97% to 82.58%, the breeding program ensured that reproductive performance remained high even as growth traits were improved. This demonstrates that with appropriate marker-assisted selection, the traditional antagonism between growth and reproduction can be overcome, enabling the development of composite breeds that combine the best characteristics of both parental lines.

### 4.6. Optimal Slaughter Age and Production Efficiency

The identification of 9 months as the optimal slaughter age for balancing meat yield and quality has important practical implications for Huang-huai sheep production. While live weight, carcass weight, and GR value increased significantly from 6 to 9 months, dressing percentage and loin muscle area showed no significant differences between the two age groups. More importantly, intramuscular fat content was significantly higher at 9 months (3.2% vs. 2.8%, *p* < 0.05) with no significant difference in shear force, indicating that the additional feeding period improved eating quality without compromising tenderness. These findings are consistent with Pewan and Güngör [36,37], who reported that extended finishing periods in sheep can enhance intramuscular fat deposition while maintaining tender texture when genetics are appropriately managed.

The increased proportion of oleic acid (C18:1n9c) and the higher UFA/SFA ratio at 9 months are also favorable from a human health perspective, as unsaturated fatty acids are associated with reduced cardiovascular disease risk [19,23]. These changes are consistent with GWAS findings linking genetic variants to carcass composition [38] and with nutritional studies showing that dietary or genetic modulation can enhance beneficial fatty acids in sheep meat [39]. The UFA/SFA ratio of 1.41 at 9 months compares favorably with values reported for other meat sheep breeds and meets consumer preferences for healthier meat products [40]. These findings suggest that extending the finishing period to 9 months not only increases carcass weight but also improves meat nutritional quality, providing a strong economic incentive for producers to adopt this production strategy.

### 4.7. Statistical Rigor and Validation of Molecular Markers

The statistical approach employed in this study addressed the reviewer’s concerns regarding multi-year and multi-farm data structure. Mixed model analysis with farm and year as random effects provided adjusted means that account for environmental variation, while EBV trend analysis confirmed the genetic basis of observed improvements. The application of Bonferroni correction to correlation analyses ensured that the strong associations between gene expression and meat quality traits were not artifacts of multiple testing. The corrected significance threshold of *p* < 0.0083, with both *CIDEa* (*r* = 0.89, *p* < 0.001) and FABP3 (*r* = 0.70, *p* < 0.001) remaining highly significant, provides confidence in the robustness of these marker-trait associations.

The detailed RT-qPCR methodology, including sample size justification (30 animals per gene), biological and technical replication, and normalization strategy, meets the standards expected for gene expression validation studies [36]. The consistency between genotype effects (Table 6), expression correlations (Figure 2 and Figure 3), and population-level phenotypic improvements (Table 5) provides triangulating evidence for the functional relevance of these markers in the Huang-huai sheep genetic background.

### 4.8. Limitations and Future Directions

Despite the significant achievements of this six-year breeding program, several limitations should be acknowledged. First, the study focused primarily on the core breeding herd, and the translation of these genetic gains to commercial production populations requires further validation. Future studies should track the performance of F1 and F2 commercial offspring to assess the consistency of trait expression across different production environments and management systems.

Second, while the “Yuyang Muxin” chip includes a comprehensive panel of functional markers, it does not cover the entire genome. As the cost of whole-genome sequencing continues to decline, integrating genome-wide association studies (GWAS) with the current chip-based approach could identify additional favorable alleles and further improve selection accuracy [26]. Recent GWAS on carcass traits [41] and investigations of myostatin introgression effects [42] illustrate the potential for discovering novel variants that could be incorporated into future versions of the chip.

Third, the absence of a formal control population, while mitigated by EBV trend analysis and statistical controls, represents a limitation inherent to commercial breeding programs. Future studies could consider maintaining a small unselected reference line to enable more precise partitioning of genetic versus environmental effects, though this must be balanced against the economic and ethical considerations of maintaining unselected animals in a production setting.

Fourth, the long-term stability of the genetic gains achieved in this program remains to be evaluated. Continuous monitoring of the core herd over multiple generations will be necessary to detect any signs of inbreeding depression or loss of genetic diversity. The maintenance of F_IS_ below 0.05 throughout the selection period is encouraging, but ongoing management of genetic diversity will be essential for the long-term sustainability of the breeding program.

Finally, the economic impact of this breeding program deserves more detailed analysis. Preliminary data from the collaborating farms suggest that the chip-based selection reduced breeding costs while improving selection accuracy, but a comprehensive cost–benefit analysis incorporating factors such as reduced generation interval, increased accuracy, improved product value, and the costs of genotyping would provide valuable guidance for other breeding programs considering adopting similar technologies. Such analysis would also help quantify the return on investment for farmers adopting genetically improved stock.

## 5. Conclusions

This six-year breeding program demonstrates that the “Yuyang Muxin” custom SNP chip is an effective tool for marker-assisted selection in Huang-huai sheep, enabling simultaneous improvement of growth, carcass, meat quality, and reproductive traits. After six years of selection, 6-month ram body weight increased by 9.1%, dressing percentage improved by 1.8 percentage points, loin muscle area expanded by 9.4%, shear force decreased by 14.1%, and intramuscular fat content increased by 40.0% (all *p* < 0.05). EBV trend analysis confirmed that these improvements were primarily attributable to genetic gain, while genetic diversity was maintained (F_IS_ < 0.05). Reproductive performance improved, with lambs weaned per ewe per year rising from 2.38 to 2.56 (*p* < 0.05). Strong correlations between *CIDEa/FABP3* expression and meat quality traits remained significant after Bonferroni correction. Nine months was identified as the optimal slaughter age. This study provides a replicable model for integrating genomic tools into sheep breeding programs.

## Figures and Tables

**Table 1:** Comparison of functional genotype frequencies in the Huang-huai Sheep core herd between 2020 and 2025.

Gene	Genotype	2020 n (%)	2025 n (%)	*p*-Value
*FecB*	BB + B+	1655 (68.97)	1982 (82.58)	<0.05
	++	745 (31.03)	418 (17.42)	
*CLPG*	CT	430 (17.92)	600 (25.00)	<0.05
	CC + TT	1970 (82.08)	1800 (75.00)	
*FABP3*	AA	960 (40.00)	1320 (55.00)	<0.05
	AG + GG	1440 (60.00)	1080 (45.00)	
*ACTC1*	CC	1104 (46.00)	1416 (59.00)	<0.05
	CT + TT	1296 (54.00)	984 (41.00)	

**Note:** Favorable genotypes associated with superior traits: *FecB* BB + B+ (high prolificacy), *CLPG* CT (muscle development), *FABP3* AA (meat quality), *ACTC1* CC (muscle development). *p*-values indicate significance of genotype frequency differences between 2020 and 2025 (Chi-square test). Total core herd size was approximately 2400 animals in each year.

**Table 2:** Comparison of body weight and body measurements of 6-month-old Huang-huai Sheep between 2020 and 2025.

Trait	Sex	2020 (n = 120)	2025 (n = 120)	*p*-Value
Body weight (kg)	♂	58.50 ± 6.55	63.80 ± 5.20	<0.05
	♀	52.45 ± 5.67	56.20 ± 4.80	<0.05
Body height (cm)	♂	71.62 ± 3.42	73.50 ± 3.10	<0.05
	♀	66.45 ± 3.85	68.20 ± 3.40	<0.05
Body length (cm)	♂	85.32 ± 5.31	88.60 ± 4.90	<0.05
	♀	75.64 ± 3.17	78.30 ± 3.50	<0.05
Chest circumference (cm)	♂	99.42 ± 7.41	104.30 ± 6.80	<0.05
	♀	94.12 ± 7.56	98.50 ± 6.90	<0.05
Cannon bone circumference (cm)	♂	9.56 ± 1.24	10.20 ± 1.10	<0.05
	♀	8.60 ± 0.88	9.10 ± 0.80	<0.05

**Note:** Data are presented as mean ± standard deviation. The 2020 population represents the baseline before selection, and the 2025 population represents the population after six years of selection using the “Yuyang Muxin” breeding chip. *p*-values indicate significant differences between 2020 and 2025 for each sex (independent samples *t*-test). All body measurements were taken at 6 months of age. The increased chest circumference and body weight in the 2025 population indicate improved meat-type conformation.

**Table 3:** Feedlot performance of 6-month-old Huang-huai sheep rams before (2020) and after (2025) six years of selection.

Group	n	ADG (g/d)	F/G (kg/kg)	Final Weight (kg)
2020 (Pre-selection)	30	295 ± 21 ^a^	4.8 ± 0.4 ^a^	54.6 ± 2.2 ^a^
2025 (Post-selection)	30	335 ± 18 ^b^	4.3 ± 0.3 ^b^	61.3 ± 2.5 ^b^

**Note:** Data are presented as mean ± SD. ADG: average daily gain; F/G: feed-to-gain ratio (total DM intake/total weight gain); **Final Weight**: body weight at the end of the 90-day feedlot trial (without fasting). Different superscript letters within the same column indicate significant differences (*p* < 0.05, independent samples *t*-test). The 2020 population represents the baseline before selection, and the 2025 population represents the population after six years of selection using the “Yuyang Muxin” breeding chip. Compared to 2020, ADG increased by 13.6% and F/G improved by 10.4%.

**Table 4:** Slaughter performance of 6-month-old Huang-huai sheep rams before (2020) and after (2025) six years of selection, with Dorper sire line as reference.

Parameter	Dorper (Sire Line) (n = 10)	2020 (Pre-Selection) (n = 30)	2025 (Post-Selection) (n = 30)
Live weight (kg)	58.2 ± 3.5	58.5 ± 6.6 ^a^	63.8 ± 5.2 ^b^
Carcass weight (kg)	32.5 ± 2.1	32.9 ± 1.6 ^a^	36.2 ± 1.8 ^b^
Dressing percentage (%)	55.8 ± 1.2	56.0 ± 1.3 ^a^	57.8 ± 1.3 ^b^
Loin muscle area (cm^2^)	24.8 ± 1.5	24.5 ± 2.1 ^a^	26.8 ± 2.1 ^b^
GR value (mm)	12.5 ± 1.2	12.8 ± 1.3 ^a^	13.2 ± 1.2 ^a^

**Note:** Data are presented as mean ± SD. Loin muscle area was measured between the 12th and 13th ribs; GR value represents tissue thickness (muscle + fat) at 11 cm from the dorsal midline between the 12th and 13th ribs. Different superscript letters within the same row indicate significant differences between 2020 and 2025 groups (*p* < 0.05, independent samples *t*-test). Dorper data are provided as reference for the paternal line and were not included in the statistical comparison; Live weight was recorded after 24 h fasting. The 2025 population showed significant improvements in live weight, carcass weight, dressing percentage, and loin muscle area compared to the 2020 baseline, approaching or exceeding the Dorper sire line values. The GR value showed no significant difference between the two years.

**Table 5:** Comparison of meat quality parameters and fatty acid composition of longissimus dorsi muscle from 6-month-old Huang-huai sheep rams before (2020) and after (2025) six years of selection.

Parameter	2020 (Pre-Selection) (n = 15)	2025 (Post-Selection) (n = 15)
**Basic meat quality**		
pH (24 h)	5.61 ± 0.04 ^a^	5.63 ± 0.05 ^a^
Meat color L* (lightness)	38.5 ± 2.1 ^a^	39.2 ± 2.0 ^a^
Meat color a* (redness)	18.2 ± 1.5 ^a^	18.8 ± 1.4 ^a^
Meat color b* (yellowness)	4.2 ± 0.6 ^a^	4.4 ± 0.5 ^a^
Shear force (N)	38.65 ± 2.10 ^a^	33.20 ± 1.90 ^b^
Intramuscular fat (%)	2.0 ± 0.3 ^a^	2.8 ± 0.4 ^b^
Drip loss (%)	2.8 ± 0.4 ^a^	2.6 ± 0.3 ^a^
Cooking loss (%)	32.5 ± 1.8 ^a^	31.8 ± 1.6 ^a^
**Fatty acid composition (% of total fatty acids)**		
C14:0 (Myristic acid)	2.8 ± 0.3 ^a^	2.6 ± 0.3 ^a^
C16:0 (Palmitic acid)	24.5 ± 1.2 ^a^	23.8 ± 1.1 ^a^
C18:0 (Stearic acid)	16.8 ± 1.0 ^a^	16.2 ± 0.9 ^a^
C18:1n9c (Oleic acid)	38.2 ± 1.5 ^a^	40.5 ± 1.4 ^b^
C18:2n6c (Linoleic acid)	8.5 ± 0.6 ^a^	9.0 ± 0.5 ^a^
C20:4n6 (Arachidonic acid)	2.2 ± 0.3 ^a^	2.6 ± 0.3 ^b^
Total SFA	44.1 ± 1.8 ^a^	42.6 ± 1.6 ^b^
Total UFA	55.9 ± 1.8 ^a^	57.4 ± 1.6 ^b^
UFA/SFA ratio	1.27 ± 0.06 ^a^	1.35 ± 0.05 ^b^

**Note:** Data are presented as mean ± SD. SFA: saturated fatty acids; UFA: unsaturated fatty acids. Meat color L* (lightness), a* (redness), and b* (yellowness) were measured using a Minolta CR-400 colorimeter; shear force was measured using a TA.XT Plus texture analyzer; intramuscular fat content was determined by Soxhlet extraction; fatty acid composition was analyzed by gas chromatography-mass spectrometry (GC-MS). Different superscript letters within the same row indicate significant differences between 2020 and 2025 groups (*p* < 0.05, independent samples *t*-test). The 2025 population showed significantly lower shear force (improved tenderness), higher intramuscular fat content, increased proportions of oleic acid (C18:1n9c) and arachidonic acid (C20:4n6), and a more favorable UFA/SFA ratio compared to the 2020 baseline.

**Table 6:** Effects of *FABP3* and *CIDEa* genotypes on meat quality traits in 6-month-old Huang-huai sheep rams (2025 population).

Gene	Genotype	n	Shear Force (N)	Intramuscular Fat (%)	C18:1n9c (%)	C20:4n6 (%)
*FABP3*	AA	16	32.1 ± 1.8 ^a^	2.9 ± 0.4 ^a^	40.8 ± 1.3 ^a^	2.7 ± 0.3 ^a^
	AG + GG	14	36.4 ± 2.0 ^b^	2.6 ± 0.3 ^b^	39.5 ± 1.2 ^b^	2.3 ± 0.3 ^b^
*CIDEa*	CC	14	33.5 ± 1.9 ^a^	3.1 ± 0.4 ^a^	40.6 ± 1.4 ^a^	2.6 ± 0.3 ^a^
	CT + TT	16	34.8 ± 2.1 ^a^	2.5 ± 0.3 ^b^	40.2 ± 1.3 ^a^	2.5 ± 0.3 ^a^

**Note:** Data are presented as mean ± SD. Different superscript letters within the same column for each gene indicate significant differences between genotypes (^a^,^b^: *p* < 0.05, independent samples *t*-test). Samples were collected from 30 six-month-old rams randomly selected from the 2025 core herd (15 from each farm). For *FABP3*, the AA genotype (favorable allele) was associated with significantly lower shear force (improved tenderness, −11.8%) and higher proportions of oleic acid (C18:1n9c) and arachidonic acid (C20:4n6). For *CIDEa*, the CC genotype was associated with significantly higher intramuscular fat content (+24.0%). These results are consistent with the gene expression correlations shown in Figure 2 and Figure 3, validating the effectiveness of these markers in the “Yuyang Muxin” breeding chip.

**Table 7:** Effect of *FecB* genotypes on reproductive performance and comparison of core herd reproductive traits before (2020) and after (2025) six years of selection.

Parameter	n	Litter Size (Lambs)	LEY (Lambs)	Lamb Survival Rate (%)
**FecB genotype effect (2025 population)**				
BB	285	1.91 ± 0.30 ^a^	-	96.2 ± 1.5 ^a^
B+	658	1.85 ± 0.42 ^a^	-	95.8 ± 1.8 ^a^
++	257	1.45 ± 0.51 ^b^	-	94.5 ± 2.1 ^a^
**Selection progress**				
2020 (Pre-selection)	1200	1.85 ± 0.28 ^A^	2.38 ± 0.14 ^A^	94.8 ± 1.6 ^A^
2025 (Post-selection)	1200	1.96 ± 0.25 ^B^	2.56 ± 0.12 ^B^	95.9 ± 1.4 ^A^

**Note:** Data are presented as mean ± SD. LEY: lambs weaned per ewe per year. Different superscript letters within the same column and section indicate significant differences: lowercase letters (^a^,^b^) for *FecB* genotype comparisons (*p* < 0.01, one-way ANOVA with Duncan’s test); uppercase letters (^A^,^B^) for selection progress comparisons between 2020 and 2025 (*p* < 0.05, independent samples *t*-test). *FecB* genotype data were obtained from 1200 ewes in the 2025 core herd. The BB and B+ genotypes showed significantly higher litter sizes compared to the ++ genotype (*p* < 0.01), confirming the favorable effect of the *FecB* mutation on prolificacy. After six years of selection, the core herd exhibited significant improvements in both litter size (+5.9%) and LEY (+7.6%) (*p* < 0.05), while lamb survival rate remained stable. The increased frequency of favorable *FecB* genotypes (from 68.97% to 82.58%, as shown in Table 1) contributed to this reproductive improvement.

**Table 8:** Comparison of slaughter performance and meat quality between 6-month and 9-month-old Huang-huai sheep rams (2025 population).

Parameter	6 Months (n = 15)	9 Months (n = 15)	*p*-Value
**Slaughter performance**			
Live weight (kg)	63.8 ± 5.2 ^a^	78.5 ± 6.1 ^b^	<0.05
Carcass weight (kg)	36.2 ± 1.8 ^a^	45.3 ± 2.2 ^b^	<0.05
Dressing percentage (%)	57.8 ± 1.3 ^a^	58.2 ± 1.4 ^a^	>0.05
Loin muscle area (cm^2^)	26.8 ± 2.1 ^a^	28.5 ± 2.3 ^a^	>0.05
GR value (mm)	13.2 ± 1.2 ^a^	15.8 ± 1.4 ^b^	<0.05
**Meat quality parameters**			
Shear force (N)	33.2 ± 1.9 ^a^	32.8 ± 1.9 ^a^	>0.05
Intramuscular fat (%)	2.8 ± 0.4 ^a^	3.2 ± 0.4 ^b^	<0.05
pH (24 h)	5.63 ± 0.05 ^a^	5.65 ± 0.05 ^a^	>0.05
Drip loss (%)	2.6 ± 0.3 ^a^	2.5 ± 0.3 ^a^	>0.05
Cooking loss (%)	31.8 ± 1.6 ^a^	31.2 ± 1.5 ^a^	>0.05
**Fatty acid composition (% of total fatty acids)**			
C18:1n9c (Oleic acid)	40.5 ± 1.4 ^a^	41.8 ± 1.3 ^b^	<0.05
C20:4n6 (Arachidonic acid)	2.6 ± 0.3 ^a^	2.7 ± 0.3 ^a^	>0.05
Total SFA	42.6 ± 1.6 ^a^	41.5 ± 1.5 ^a^	>0.05
Total UFA	57.4 ± 1.6 ^a^	58.5 ± 1.5 ^a^	>0.05
UFA/SFA ratio	1.35 ± 0.05 ^a^	1.41 ± 0.05 ^b^	<0.05

**Note:** Data are presented as mean ± SD. Different superscript letters within the same row indicate significant differences between age groups (^a^,^b^: *p* < 0.05, independent samples *t*-test). Samples were collected from 30 six-month-old and 30 nine-month-old rams randomly selected from the 2025 core herd (15 from each farm per age group). GR value represents tissue thickness at 11 cm from the dorsal midline between the 12th and 13th ribs. SFA: saturated fatty acids; UFA: unsaturated fatty acids. While live weight, carcass weight, and GR value increased significantly with age (*p* < 0.05), dressing percentage and loin muscle area showed no significant differences between the two age groups. Importantly, intramuscular fat content was significantly higher at 9 months (*p* < 0.05) with no significant difference in shear force (tenderness), indicating that meat quality was maintained or improved with extended feeding. The proportion of oleic acid (C18:1n9c) and the UFA/SFA ratio were also significantly higher at 9 months (*p* < 0.05). These results demonstrate that 9 months of age represents the optimal slaughter age for balancing meat yield and quality in Huang-huai Sheep.

## Data Availability

The original contributions presented in this study are included in the article. Further inquiries can be directed to the corresponding authors.
